# Endophyte-Infected Tall Fescue Affects Rumen Microbiota in Grazing Ewes at Gestation and Lactation

**DOI:** 10.3389/fvets.2020.544707

**Published:** 2020-10-14

**Authors:** Jianmin Chai, Saleh Alrashedi, Ken Coffey, Joan M. Burke, Kristina Feye, Steven C. Ricke, Si Hong Park, J. Lannett Edwards, Jiangchao Zhao

**Affiliations:** ^1^Department of Animal Science, Division of Agriculture, University of Arkansas, Fayetteville, AR, United States; ^2^United States Department of Agriculture, Agricultural Research Service, Booneville, AR, United States; ^3^Department of Food Science and Center for Food Safety, University of Arkansas, Fayetteville, AR, United States; ^4^Department of Food Science and Technology, Oregon State University, Corvallis, OR, United States; ^5^Department of Animal Science, University of Tennessee, Knoxville, TN, United States

**Keywords:** tall fescue, toxins, microbiota, rumen microbiota, sheep, ergot alkaloids, endophyte

## Abstract

Tall fescue (*Schedonorus arundinaceus*) is a cool-season perennial grass that is widely used as a forage for many livestock species including sheep. An endophyte (*Neotyphodium coenophialum)* in tall fescue produces ergot alkaloids that enhance plant survival but produce toxicosis in animals. The objective of this study was to investigate the rumen microbiota from gestation and lactation in ewes grazing tall fescue pastures with high (HA) or moderate (MA) levels of endophyte infection, and their relationship with serum parameters. Data were collected at the beginning of the study (d1), the week before initiation of lambing (d51), and at the end of the trial (d115). The rumen microbiota was evaluated using 16S rRNA gene sequencing. Ewes grazing HA had greater serum non-esterified fatty acid (NEFA) (*P* = 0.024) levels compared with ewes in MA pasture at d115. Both the number of observed OTUs and Shannon diversity index tended (*P* = 0.08, *P* = 0.06) to be greater for HA than for MA on d115. At the genus level, *Prevotella* relative abundance increased with time in both MA and HA (on d1, d51, and d115: 15.17, 25.59, and 24.78% in MA; 14.17, 18.10, and 19.41% in HA). Taxa unclassified at the genus level including (unclassified) *Lachnospiraceae, Coriobacteriaceae*, and *Veillonellaceae* exhibited higher abundances in HA at d51 (3.72, 2.07, and 11.22%) compared with MA (2.06, 1.28, and 7.42%). The predictor microbiota for HA and MA were identified by a random forest classification model. The HA predictors included bacteria associated with unclassified *Coriobacteriaceae* and *Ruminococcaceae*. Other OTUs classified as *Prevotella* and *Clostridiales* could be microbial predictors for MA. The OTUs classified as *Prevotella* and *Lachnospiraceae* were negatively correlated with serum concentration of prolactin. Negative correlations with NEFA were observed in the microbiota such as species affiliated to unclassified *Clostridiales* and *Prevotella*. OTUs classified as *Bacteroidetes* and *Coriobacteriaceae* exhibited a positive correlation with NEFA. Our study confirmed that the rumen microbiota populations were affected by high levels of toxins in endophyte-infected tall fescue and were associated with host hormone and energy metabolism.

## Introduction

Tall fescue [*Schedonorus arundinaceus* (Schreb.) Dumort, syn. *Festuca arundinacea*] is a cool-season perennial grass that has been widely used as a forage for livestock (1). A symbiotic association between tall fescue and the fungus *Neotyphodium coenophialum* has been detected in more than 90% of tall fescue pastures (2), which increases survival of tall fescue grass during extreme weather conditions (3, 4). However, the fungal endophyte produces a range of mycotoxins that can be detrimental to livestock performance (5, 6). One of the most common classes of mycotoxins associated with *N. coenophialum* include ergot alkaloids, in which toxicity is typically characterized within livestock by reduced weight gain and volatile fatty acid absorption, restricted blood flow, and reduced reproductive rates (7, 8). Ergovaline is the main ergopeptine alkaloid produced in the endophyte-infected tall fescue, and is considered the primary causative agent of fescue toxicosis in livestock (9, 10). It has been proposed that mechanistically, the structure of ergot alkaloids enables these compounds to act as sympathetic monoamine mimetics for host receptors (11). Ergot alkaloids contain a tetracyclic ergoline ring and are structurally similar to biogenic amines such as serotonin, dopamine, norepinephrine, and epinephrine (11). This structural confirmation enables the compound to ligate receptors for biogenic amines, which have been hypothesized to decrease serum prolactin (PRL) concentrations and induce vasoconstriction via downstream signaling events (12, 13). In addition, the EC_50_ dose of ergovaline is relatively low, and can induce a series of adverse reactions in animals, such as the reduction of feed efficiency and gastrointestinal passage rates (7).

Previous research has focused on elucidating the effects of endophyte-infected tall fescue on performance and other physiological parameters, but few studies have been conducted to evaluate its effects on the rumen microbiota. It is important to evaluate the effects of these bioactive alkaloids on the rumen and its microbial population, as the microbial population directly aids in providing nutrients to the ruminant host by forage fermentation (i.e., tall fescue) and also impacts lactation energy requirements (14, 15). Rumen microorganisms are able to degrade many of the ergot alkaloids consumed by the hosts; however, the identification of the organisms responsible for toxicity degradation is unknown (16). In an *in vitro* study conducted by Harlow et al. (17), it was determined that ruminal ammonia-hyperproducing bacteria *Peptostreptococcus anaerobius* and *Clostridum* species *sporogenes, sticklandii*, and *aminophilum* as well as *Prevotella bryantii*, albeit to a lesser extent, contribute to the breakdown and metabolism of ergovaline. However, there remains a substantial information gap between the *in vitro* studies done with the pure bacterial cultures mentioned above and how they may contribute to ergovaline detoxification in the animal. Moreover, little is known regarding potential associations between the *in vivo* rumen microbiota and the performance and well-being of pregnant ewes grazing tall fescue. Accordingly, the objectives of this study were to evaluate changes to the rumen microbiota from gestation to lactation in ewes grazing tall fescue with high or moderate levels of toxic endophyte infection, and its association with serum parameters.

## Materials and Methods

This study was conducted from early February to late May 2016 at the USDA, ARS Dale Bumpers Small Farms Research Center (DBSFRC), Booneville, AR, USA. All experimental procedures and husbandry practices in this experiment were reviewed and approved by the University of Arkansas Institutional Animal Care and Use Committee (Protocol # 16046) and the USDA ARS Institutional Animal Care and Use Committee.

### Animal Management and Samples Collection

Fifty Katahdin ewes (ages 2–9 years) confirmed as pregnant via ultrasound were selected from the DBSFRC flock, then stratified by body weight (BW) within age and the number of fetuses and were allocated randomly into three groups. Groups 1 and 3 contained 15 ewes each, with group 2 containing 20 ewes. Groups 1 and 3 were allocated to tall fescue pastures with a high level of endophyte infection (HA; 90% infection), and group 2 was allocated to a tall fescue pasture with moderate endophyte infection (MA; 58% infection). The numbers (58 and 90%) are derived from collecting individual tillers from each pasture (*n* = 50) and subjecting each individual tiller to the immunoblot test kit that is available commercially. This kit is a presence/absence test for endophyte infection and is widely accepted as being accurate in the determination of the presence of *N. coenophialum*. This infection was then verified by quantitative analysis of ergovaline and total ergot alkaloids to provide an indication of the level of toxicity of the different pastures. Within each of these larger groups, ewes were selected at random for rumen sampling. This resulted in five ewes selected from group 1 (HA) and three ewes from group 3 (HA) with an average initial BW of 51.6 ± 2.64 kg and an initial average body condition score (BCS; 1 = emaciated and 5 = obese) of 2.8 ± 0.18. Seven ewes were selected randomly from group 2 (MA) with an average BW of 40.6 ± 2.82 kg, and an initial BCS of 2.7 ± 0.19. All ewes had unrestricted access to water and free choice to trace mineralized salt (Supplementary Table 1).

Blood samples were taken from the jugular vein of each ewe directly prior to day 1 (d1), the week before initiation of lambing (d51), and at the end of the trial (d115), which corresponded to ~60 d post-parturition. Blood was collected into serum separator vacuum tubes (BD #367988 Vacutainer®, Becton Dickinson, Inc., Franklin Lakes, NJ, USA). Samples were transported on ice and stored in a standard refrigerator at 4°C overnight. The samples were centrifuged (3,000 × g for 20 min.), followed by transfer to plastic tubes, and stored frozen (−20 °C) for subsequent analyses.

Rumen fluid samples (~10 mL) were taken directly from the rumen from all ewes at d1, d51, and d115 by orogastric intubation using sterile 60 mL syringes and autoclaved tubes. Rumen samples from each ewe were kept separately in 50 mL centrifuge tubes and transported on dry ice to the University of Arkansas, and stored at −80°C until DNA extraction.

Tall fescue grass samples were collected monthly from each pasture throughout the study. Samples of tall fescue (*n* = 20/pasture) were harvested by walking in a zigzag pattern through each pasture and hand-clipping random samples to a 2.5 cm stubble height. The samples were placed in zip-lock plastic bags, stored on ice immediately, and then moved to a conventional freezer for at least 2 h. The grass samples were transported to the University of Arkansas Animal Science Department, and stored in an ultra-low freezer (−80°C), until they were lyophilized. Dried forage samples were ground to pass through 1-mm screen using a Thomas-Wiley laboratory mill model 4 (Arthur Thomas Co. Philadelphia, PA, USA) and stored in an ultra-low freezer except during analyses. Tillers for testing for the presence of endophyte fungus were collected on February 2nd by walking a zigzag pattern in the pastures, and collecting tall fescue tiller samples at random (50 per pasture). Stems were cut with a sharp knife to a height of 2.5 cm above of the soil surface. Tissue that contained the leaves was discarded; the tillers were rolled into moist paper towels to prevent drying, and then placed in plastic zip lock bags and stored in a refrigerator (1°C) pending endophyte analysis.

### Measurements of Forage and Serum

Sequential analyses for forage neutral detergent fiber (NDF) and acid detergent fiber (ADF) were performed using the filter bag procedures described by Vogel et al. (18) using an Ankom 200 Fiber Analyzer (ANKOM Technology Corporation, Fairport, NY, USA). Nitrogen concentrations were determined via the Dumas total combustion method (Elmentar Americas, Mt. Laurel, NJ, USA; Method 990.03) (19). Crude protein (CP) was calculated by multiplying the total N concentration by 6.25. Total ergot alkaloids were determined for using Agrinostics Photo screen Ergot Alkaloids kit (Agrinostics Ltd. Co., Watkinsville, GA, USA) (20). Forage ergovaline concentrations were also determined using the HPLC procedure of (21). Pasture endophyte infection rates were analyzed using immunoblot test kits (Agrinostics Ltd. Co., Watkinsville, GA, USA).

Serum samples were analyzed for non-esterified fatty acid (NEFA) concentrations using an *in vitro* enzymatic colorimetric kit [NEFA-HR (2); Wako Chemicals, Inc., Richmond, VA, USA]. Serum prolactin (PRL) concentrations were determined at the University of Tennessee using their standard lab protocol following the procedures of Bernard et al. (22) with inter- and intra-assay coefficients of variability (CV) of 4.24 and 5.69%, respectively.

### DNA Extraction and Next-Generation Sequencing

Total bacterial DNA was extracted using a PowerSoil^TM^ DNA Isolation Kit (MO BIO Laboratories, Inc. Carlsbad, CA, USA) based on the manufacturer's instructions. Moreover, a bead beating step for rapid and thorough homogenization of bacterial cells was incorporated to yield high quantity and quality rumen microbial DNA. DNA concentrations were measured using a NanoDrop (Thermo Fisher Scientific, Waltham, MA, USA). The V4 region of bacterial 16S rRNA gene was amplified for Illumina sequencing with the following primers 5′-GTGCCAGCMGCCGCGGTAA-3′ (forward) and 5′-GGACTACHVGGGTWTCTAAT-3′ (reverse) (23). PCR was performed using a T100 thermal cycler (Bio-Rad, Hercules, CA, USA) with the following conditions: 30 s initial denaturation at 95°C; 30 cycles at 95°C for 10 s annealing at 55°C for 30 s, and at 72°C for 1 min; at a 72°C final extension for 10 min. Two of microliters amplicons were examined on 1.0% agarose gel and remaining PCR products were subsequently normalized using a SequalPrep^TM^ Normalization kit (Life Technology, Carlsbad, CA, USA) according to the manufacturer's recommendation. A 10 ng DNA aliquot was utilized to construct a sequencing library following a previous report (24). The concentration and quality of the library were measured using a KAPA Library Quantification Kit (Kapa Biosystem, Woburn, MA, USA) via a quantitative PCR (qPCR, Eppendorf, Westbury, NY, USA) assay and an Agilent 2100 Bioanalyser System (Agilent, Santa Clara, CA, USA), respectively. The library was mixed with PhiX control v3 (5%, v/v) (Illumina), loaded on a MiSeq v2 (2 × 250 bp, 500 cycle) reagent cartridge and then sequenced on an Illumina MiSeq sequencer (Illumina, San Diego, CA, USA) for next-generation sequencing.

### Bioinformatics

The MOTHUR (v.1.39.1) software package was used to process raw sequences (25) following the standard procedures for MiSeq sequences (24). The forward and reverse sequences were first merged by using the make.contigs command in MOTHUR. Low-quality sequence reads were removed based on homopolymers longer than 8 bp, ambiguous bases, and raw reads of 500 bp or longer. The sequences were aligned with the SILVA database release 128 which contained the full-length 16S rRNA gene sequences (https://www.mothur.org/wiki/Silva_reference_files#Release_128). The chimeric sequences were removed using UCHIME (26). Also, singletons were removed to decrease the number of spurious sequences. High-quality sequences were clustered into operational taxonomic units (OTUs) having a similarity of 97% and classified using the RDP Classifier, with a naïve Bayesian algorithm (27). After normalization to the smallest number of reads (6946), alpha and beta diversity were estimated. Alpha diversity metrics consisted of the number of observed OTUs, and the Shannon diversity index. Bray-Curtis and Jaccard distance metrics were determined to compare the dissimilarities in community structure and membership, respectively. Principal Coordinate Analysis (PCoA) was utilized to visualize these distances. Analysis of similarity (ANOSIM) was also conducted in MOTHUR for testing the statistical significance of beta diversity distances. The raw sequences in the current study are available in the NCBI Sequence Read Archive repository (project number PRJNA577241).

### Statistical Analyses

Based on a completely randomized design, BW, BCS, PRL, NEFA, and alpha diversities were tested using a linear mixed-effect model in R (v 3.6.0). Treatment and sampling time were included as fixed effects, while both individual dams, which served as experimental units, and the number of lambs born of each dam were included as nested random effects. The default unstructured covariance structure was used in linear mixed model when performing the “lmer” function (“lme4” package of R). *P*-values lower than 0.05 were considered as significant.

To analyze rumen microbiotas for differentiating high and moderate endophyte infection, a random forest model (area under the receiver operating characteristic curve (AUC) of the random forest, AUC-RF) with 10-fold cross-validation was performed (28). Thus, the model predicted the left-out subject and results were plotted as Receiver Operator Characteristic curves using the pROC package (v.1.13). The optimal predictors of AUCRF were listed based on their mean decrease accuracy (MDA). The boxplots portraying the relative abundance of optimal predictors was performed using the R “ggplot2” package (v.3.0) and labeled *p*-values of Wilcoxon test. To estimate the relationship between the microbiota and the animal phenotype (serum concentration of PRL and NEFA), a regression-based random forest model was performed using the package “randomForest” v.4.6.-7 in R with 10,000 trees. Top 50 predictors were selected and their relative abundances were then analyzed by using Pearson correlation with PRL and NEFA, respectively.

## Results

### Forage Characteristics and Ewes' Phenotype

The ergovaline concentration in both treatments ranged from 55 to 243 μg/kg during the experiment (February to May 2016, Booneville, AR, USA), which did not coincide with ambient temperatures and precipitation (Table 1 and Supplementary Table 2). Ergovaline concentrations were lowest in both treatments at the beginning of the study and increased gradually, resulting in the greatest concentrations at the end of the study in both HA and MA. The total ergot alkaloid concentrations also exhibited a similar pattern. While the concentration of ergovaline and total ergot alkaloids changed over time, they were greater in HA than MA throughout this study. Although ergovaline concentrations were low (<100 μg/kg) from both treatments on the first three sampling dates, the concentrations were 5.5, 1.5, 1.1, and 2.3 times greater from HA compared with MA on the February (2), March (22), April (12), and May (25) sampling dates, respectively. Total ergot alkaloids followed a somewhat similar pattern to ergovaline concentrations, excluding the fact that the lowest total ergot alkaloid concentrations occurred on the March (22) sampling date. Composition of other nutrients including crude protein and fiber was not appreciably different between the two treatments throughout this study.

**Table 1:** Forage quality components and ergot alkaloid concentrations from tall fescue pastures that had moderate (MA) and high (HA) *N. coenophialum* infection levels and grazed by gestation and lactation ewes.

**Date**	**Days**	**Forage**	**CP**,	**ADF**,	**NDF**,	**Ergovaline**,	**Total Ergot**
			**%**	**%**	**%**	**μg/kg**	**Alkaloids**,
							**μg/kg**
Feb 2, 2016	1	HA	10.0	24.0	40.5	55	2,471
		MA	11.6	21.7	36.1	10	586
Mar 22, 2016	50	HA	11.6	34.5	57.3	60	717
		MA	11.7	30.4	51.6	40	376
Apr 12, 2016	71	HA	15.6	28.3	48.3	63	1,128
		MA	16.9	29.3	49.4	55	811
May 25, 2016	114	HA	16.8	31.1	51.2	243	3,130
		MA	18.5	29.8	49.1	105	1,314

*CP, crude protein; ADF, acid detergent fiber; NDF, neutral detergent fiber*.

The BW and body condition scores of ewes during this study were not different (*P* > 0.10) between the HA and MA treatment groups, but these measurements decreased with sampling time (*P* < 0.05) (Table 2). Serum PRL and NEFA concentrations were not different (*P* > 0.10) between treatments throughout the study (Supplementary Table 3). However, they were affected by sampling time (*P* < 0.05). Moreover, serum NEFA displayed an interaction between treatment and sampling time (*P* < 0.05). NEFA concentration were higher on d51 than on d1 and d115 (*P* < 0.05). Serum NEFA was not different (*P* > 0.10) between treatments on d1 and d51, while ewes grazing HA had higher NEFA (*P* = 0.024) on d115 compared with ewes on MA pasture.

**Table 2:** Growth performance.

	**HA**	**MA**	**SEM^**a**^**	***P*****-values**		
				**Treatment**	**Sampling**	**Treatment ×**
					**time**	**Sampling time**
**BW/kg**						
d1	50.1	47.6	1.95	0.420	<0.001	0.282
d51	48.7	47.8	2.18			
d115	47.6	44.2	1.97			
**Body condition scores**^**b**^						
d1	2.8	2.9	0.12	0.913	<0.001	0.405
d51	2.3	2.4	0.14			
d115	2.2	2.1	0.32			

Body weight (BW) and body condition scores from ewes grazing tall fescue pastures with high (HA) and moderate (MA) N. coenophialum infection from February through May. Lambing began on d51 and d115 corresponded to the end of the study when lambs averaged 60 days of age.

a SEM = Pooled standard error of the means.

b *Body condition scores were on a scale from 1 to 5 where 1 is emaciated and 5 is obese*.

### Alpha and Beta Diversities of the Rumen Microbiota

The microbial diversity in ruminal fluid between ewes grazing HA and MA endophyte tall fescue within different periods was initially investigated. Sampling time affected alpha diversity measures (*P* < 0.05) (Table 3), however, no treatment effects or interaction with sampling time was observed (*P* > 0.05). Overall, rumen microbial diversity decreased on lambing days (d51) and then reached the initial level on the lambs' weaning day (d115).

**Table 3:** Alpha diversities of rumen microbiota in ewes grazing tall fescue pastures with high (HA) and moderate (MA) *N. coenophialum* infection.

	**HA**	**MA**	**SEM^**a**^**	***P*****-values**		
				**Treatment**	**Sampling**	**Treatment ×**
					**time**	**Sampling time**
**Shannon index**						
d1	6.0	6.0	0.05	0.414	<0.001	0.355
d51	5.7	5.5	0.09			
d115	6.0	5.9	0.04			
**Observed OTUs**						
d1	1302.7	1282.7	24.79	0.321	<0.001	0.453
d51	1060.3	974.2	48.17			
d115	1241.5	1143.9	22.73			

a *SEM = Pooled standard error of the means*.

**Figure 1:**
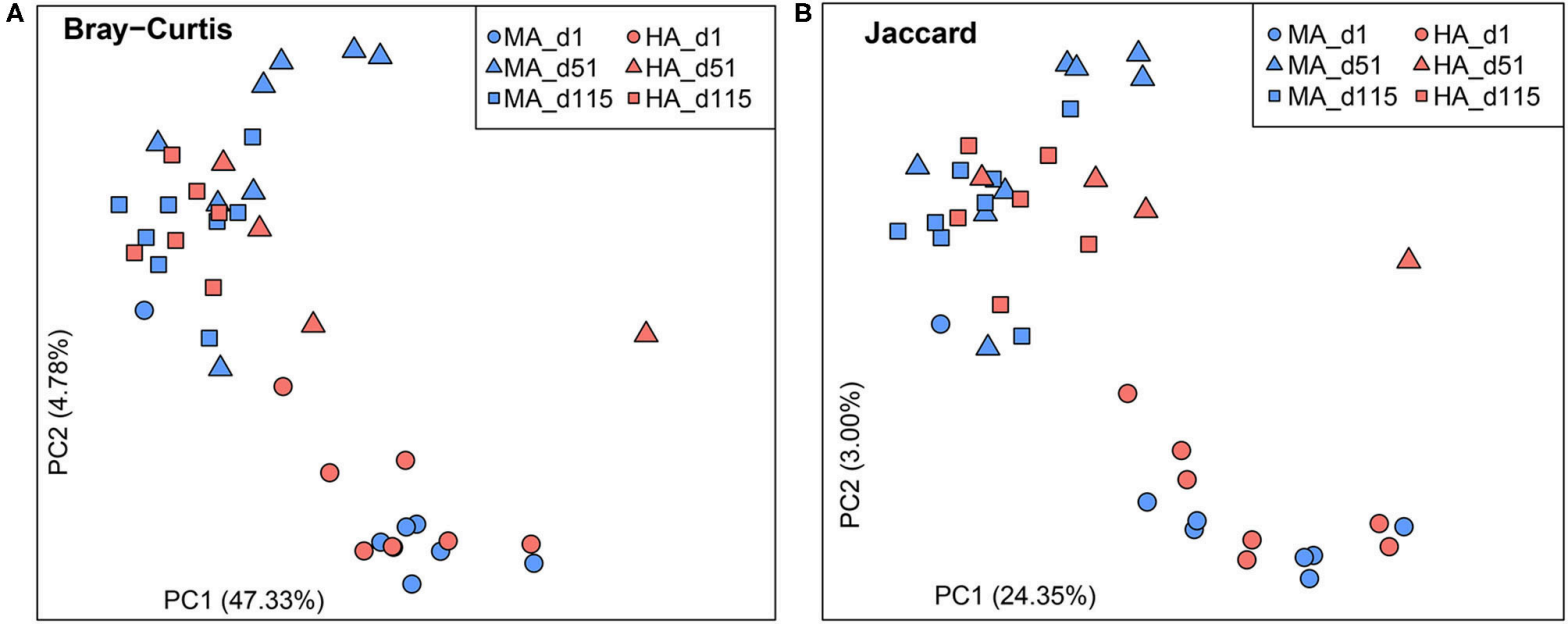
Beta diversity plots of rumen microbiota in ewes grazing tall fescue pastures with high (HA) and moderate (MA) *N. coenophialum* infection. **(A,B)** Principal coordinate analysis (PCoA) plots based on the Bray-Curtis and Jaccard similarities. The treatments of MA and HA are differentiated by colors (blue and red), and sampling days (d1, d51 and d115) are distinguished by shape (circle, triangle, and square, respectively).

Bray-Curtis and Jaccard similarities were calculated for beta diversity (Figures 1A,B). No differences were observed on d1 based on Bray-Curtis and Jaccard similarities (ANOSIM: *R* = −0.03, 0.03; *P* = 0.58, 0.35). On d51, the rumen microbial community structure and membership tended to be distinct between HA and MA (ANOSIM: *R* = 0.30, 0.29; *P* = 0.06, 0.07). In the lactation period (d115), rumen microbiota were different between ewes grazing HA and MA tall fescue (ANOSIM: *R* = 0.28, 0.31; *P* = 0.02, 0.01). In addition, d1 samples were distinct from those of the other two time points (d51 and d115).

### Taxonomic Composition of the Rumen Microbiota

**Figure 2:**
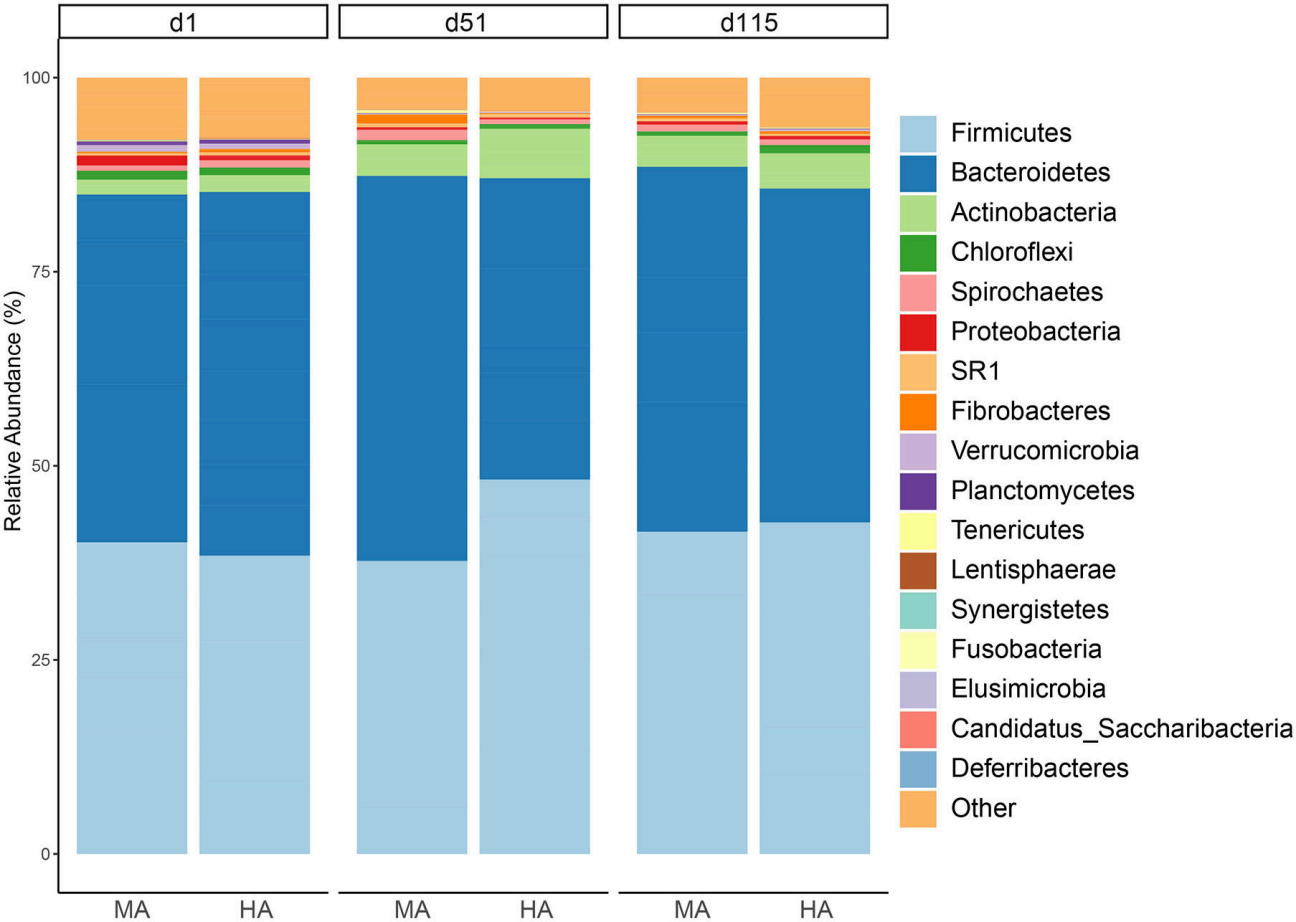
Relative abundance of rumen phyla from ewes consuming tall fescue infected with moderate (MA) or high (HA) levels of *N. coenophialum* on days 1 (d1), 51 (d51), and 115 (d115). Average relative abundance for each phylum by treatment and sampling time. Each color represents the relative abundance of a bacterial taxon on the stacked bar chart.

*Bacteroidetes* and *Firmicutes* (43.26 and 39.86% across all samples) were the dominant phyla in the rumen fluid (Figure 2). In this study, the relative abundances of the *Bacteroidetes* in MA were 42.51, 47.95, and 45.39% on d1, d51, and d115, while its relative abundances in HA were 44.68, 37.58, and 41.44% on d1, d51, and d115, respectively. On d51, the phylum *Firmicutes* was less relatively abundant in MA pasture (36.51%) compared with HA pastures (46.70%). The *Actinobacteria* phylum exhibited a similar pattern with *Firmicutes*, and its relative abundance on d51 were 3.94 and 6.16% in MA and HA, respectively.

**Figure 3:**
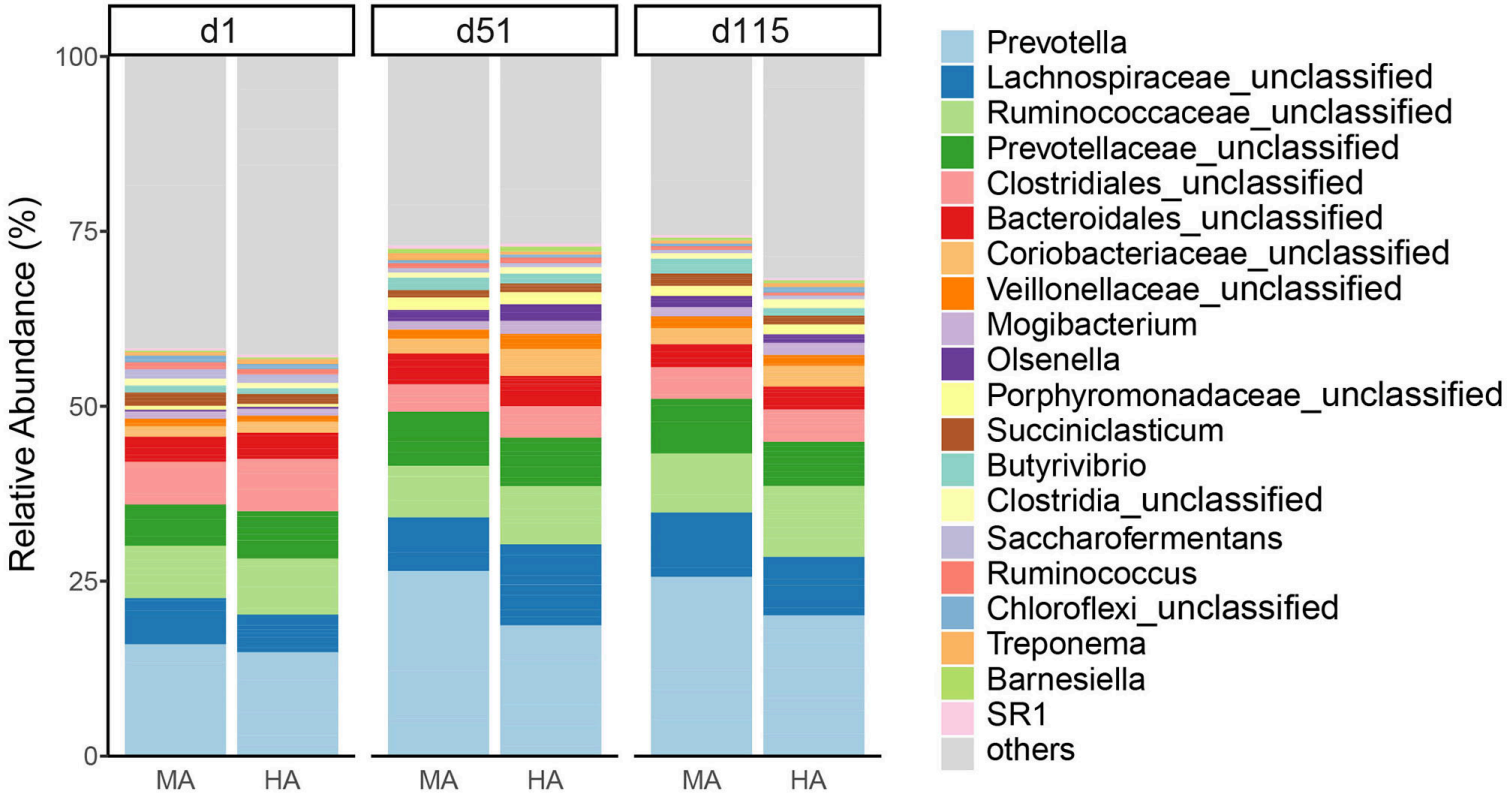
Relative abundance of rumen bacteria (genus) from ewes consuming tall fescue infected with moderate (MA) or high (HA) levels of *N. coenophialum* on days 1 (d1), 51 (d51), and 115 (d115). Each color represents the relative abundance of a bacterial taxon on the stacked bar chart.

At the genus level (Figure 3), the dominant bacteria were *Prevotella* (19.54%) across all samples, followed by unclassified *Lachnospiraceae* (7.94%), unclassified *Ruminococcaceae* (7.62%), and unclassified *Prevotellaceae* (6.46%). The abundances of *Prevotella* increased with time in both MA and HA groups (on d1, d51 and d115: 15.17, 25.59, and 24.78% in MA; 14.17, 18.10, and 19.41% in HA). The unclassified *Lachnospiraceae* in HA were in higher abundance (11.22%) on d51 compared with MA (7.42%). Other minor genera such as unclassified *Coriobacteriaceae* and unclassified *Veillonellaceae* increased with time, and also had greater abundances in HA at d51 (3.72 and 2.07%) compared with MA (2.06 and 1.28%). Unclassified *Ruminococcaceae* on d115 were 9.78 and 8.13% in HA and MA groups, respectively.

### Differential Rumen Microbiota Between Ewes Grazing HA and MA Tall Fescue Forage

**Figure 4:**
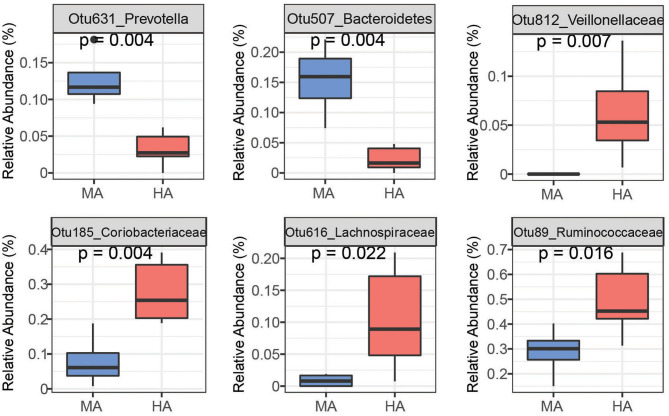
Bacteria OTUs determined using random forest differentiating MA and HA on d51. The rumen microbial data were tested using the Kruskal-Wallis test. MA and HA represent tall fescue with moderate or high levels of *N. coenophialum*, respectively.

**Figure 5:**
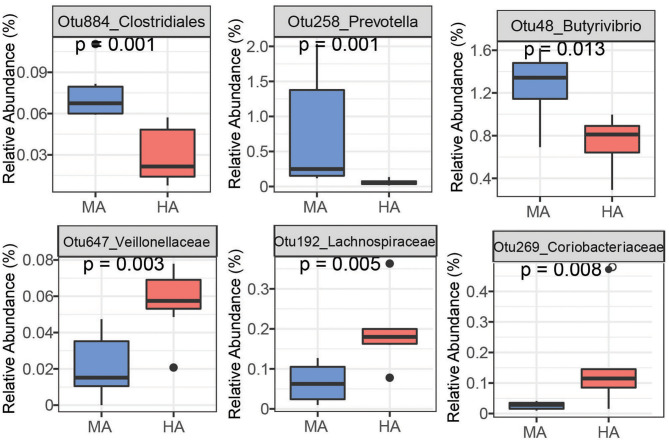
Bacteria OTUs determined using random forest differentiating MA and HA on d115. The rumen microbial data were tested using the Kruskal-Wallis test. MA and HA represent tall fescue with moderate or high levels of *N. coenophialum*, respectively.

Random Forest is able to analyze high dimensional microbiota data that usually has more variables (e.g., OTUs) than samples with complicated interactions between OTUs, and therefore, has been widely used to analyze human and animal microbiome data. Using random forest, a set of bacterial OTUs that differentiated HA and MA ewes were identified. The top 25 predictors are listed based on their ability to differentiate between ewes grazing HA and MA tall fescue (Supplementary Figures 2, 3). Six among the top 25 OTUs that differentiate HA and MA were listed as the most sensitive in distinguishing between the forages (Figures 4, 5).

On d51, the rumen species can differentiate MA and HA accurately by yielding AUC = 1 (sensitivity = 1, specificity = 1) (Supplementary Figure 1A). Several OTUs classified as *Prevotella* (Otu631, Otu134, OTU457, Otu266, and Otu11), *Fibrobacter* (Otu522), and unclassified *Ruminococcaceae* (Otu345) exhibited greater abundance in MA (Figure 4 and Supplementary Figure 2). Other OTUs including unclassified *Veillonellaceae* (Otu812), unclassified *Coriobacteriaceae* (Otu185, Otu269), unclassified *Lachnospiraceae* (Otu262, Otu616 and Otu23), and unclassified *Ruminococcaceae* (Otu89) were enriched in the HA treatment. Moreover, several OTUs classified as *Prevotella* occurred in higher abundance in HA, including Otu540, Otu717, and Otu272.

On d115, high accuracy in the random forest model was observed (Supplementary Figure 1B). Ewes grazing MA had high abundances of OTUs affiliated with unclassified *Prevotella* (Otu58, Otu171, Otu238), *Clostridiales* (Otu884), *Bacteroidetes* (Otu599 and Otu1102), *Butyrivibrio* (Otu48 and Otu215) and *Succiniclasticum* (Otu36) (Figure 5 and Supplementary Figure 3). Other OTUs enriched in HA were associated with unclassified *Veillonellaceae* (Otu647), unclassified *Coriobacteriaceae* (Otu269), unclassified *Lachnospiraceae* (Otu192), unclassified *Ruminococcaceae* (Otu49 and Otu345), and *Mogibacterium* (Otu10).

### The Association Between the Ewe Rumen Microbiota and Serum PRL

To better understand the association between the rumen microbiota and serum PRL, a random forest regression model using PRL concentration as outcomes and the top 500 OTUs as independent variables was generated. Pearson correlations were subsequently estimated among the selected top 50 bacterial abundances and PRL. The bacteria with significant correlations are listed in Table 4. Some OTUs such as *Lachnospiraceae* (Otu896) exhibited a negative relation with PRL in both gestation and lactation periods (d51: *r* = −0.64, *p* = 0.03; d115: *r* = −0.52, *p* = 0.06).

**Table 4:** The significant Pearson relationship between top bacteria OTUs identified by random forest regression model and serum prolactin (PRL) concentrations from ewes grazing *N. coenophialum* infected tall fescue pastures.

**d51**	***r***	***p***	**d115**	***r***	***p***
**Negative correlations**			**Negative correlations**		
Otu353_Coriobacteriaceae	−0.80	0.003	Otu126_Bacteroidales	0.66	0.010
Otu376_Prevotella	−0.79	0.003	Otu483_Lachnospiraceae	0.66	0.011
Otu35_Prevotella	−0.76	0.007	Otu828_Bacteroidetes	0.66	0.011
Otu253_Bacteroidetes	−0.75	0.007	Otu257_Prevotella	0.65	0.012
Otu60_Prevotella	−0.75	0.008	Otu220_SR1	0.58	0.031
Otu420_Prevotella	−0.71	0.015	Otu502_SR1	0.58	0.031
Otu884_Clostridiales	−0.70	0.017	Otu464_Anaerovibrio	0.56	0.036
Otu470_Bacteroidetes	−0.70	0.017	Otu379_Prevotella	0.54	0.044
Otu601_Prevotellaceae	−0.70	0.017	Otu878_Bacteroidetes	0.54	0.045
Otu920_Ruminococcaceae	−0.69	0.018	Otu896_Lachnospiraceae	0.52	0.057
Otu398_Prevotella	−0.68	0.022			
Otu222_Bacteroidetes	−0.68	0.022			
Otu904_Prevotellaceae	−0.65	0.030			
Otu467_Prevotella	−0.65	0.030			
Otu134_Prevotella	−0.65	0.031			
Otu896_Lachnospiraceae	−0.64	0.034			
Otu266_Prevotella	−0.64	0.034			
Otu978_Lachnospiraceae	−0.64	0.035			
Otu614_Prevotella	−0.63	0.037			
**Positive correlation**			**Positive correlation**		
Otu321_Olsenella	0.59	0.051	Otu978_Lachnospiraceae	0.56	0.039
Otu674_Coriobacteriaceae	0.60	0.049	Otu762_Ruminococcaceae	0.58	0.031
Otu180_Mogibacterium	0.61	0.048	Otu20_Olsenella	0.59	0.027
Otu745_Clostridiales	0.61	0.044	Otu121_Clostridia	0.61	0.021
Otu786_Bacteroidales	0.66	0.027	Otu825_Bacteroidetes	0.61	0.020
Otu526_Porphyromonadaceae	0.69	0.019	Otu912_Firmicutes	0.70	0.005
Otu264_Chloroflexi	0.69	0.018			

On d51, most OTUs classified as *Prevotella* were negatively correlated with PRL, e.g., Otu134 and Otu266 (*r* = −0.65, −0.64; *p* = 0.03, 0.03). Other OTUs belonging to unclassified *Bacteroidetes* (Otu253, Otu470, and Otu222), unclassified *Clostridiales* (Otu884), unclassified *Ruminococcaceae* (Otu920), and *Coriobacteriaceae* (Otu353) were also negatively correlated. In contrast, unclassified *Coriobacteriaceae* (Otu674), *Mogibacterium* (Otu180), *Clostridiales* (Out745), *Bacteroidetes* (Otu786), *Porphyromonadaceae* (Otu526), and unclassified *Chloroflexi* (Otu264) were positively correlated with PRL.

On d115, Otu257, and Otu379 classified as *Prevotella* were negatively correlated with PRL. Negative relationships were also observed in unclassified *SR1* (Otu220 and Otu502), unclassified *Bacteroidetes* (Otu126, Otu828, Otu878), and *Anaerovibrio* (Otu464). Conversely, when serum PRL concentration increased, the abundances of unclassified *Lachnospiraceae* (Otu978), unclassified *Ruminococcaceae* (Otu762), *Olsenella* (Otu20), and unclassified *Clostridia* (Otu121) increased linearly.

### The Correlation of Bacterial OTUs in the Ewe Rumen and Serum NEFA

To examine the relationship between serum NEFA and rumen microbiota we developed random forest regression models and performed Pearson correlations analysis, which shows the significant correlations between microbial abundances and serum NEFA in Table 5. Interestingly, the common OTU555 (unclassified *Clostridiales*) was negatively correlated with NEFA on d51 (*r* = −0.71, *p* = 0.01), while a positive correlation on d115 (*r* = 0.54, *p* = 0.05) was found.

**Table 5:** The significant Pearson relationship between top bacteria OTUs identified by random forest regression model and serum non-esterified fatty acid (NEFA) from ewes grazing *N. coenophialum* infected tall fescue pastures.

**d51**	***r***	***p***	**d115**	***r***	***p***
**Negative correlation**			**Negative correlation**		
Otu555_Clostridiales	−0.71	0.009	Otu238_Prevotellaceae	−0.60	0.024
Otu475_Prevotella	−0.66	0.020	Otu479_Clostridiales	−0.55	0.043
Otu780_Clostridiales	−0.64	0.024	Otu599_Bacteroidetes	−0.46	0.096
Otu551_Coriobacteriaceae	−0.63	0.027			
Otu970_Prevotella	−0.59	0.042			
Otu505_Prevotella	−0.58	0.059			
**Positive correlation**			**Positive correlation**		
Otu438_Bacteroidales	0.64	0.024	Otu555_Clostridiales	0.54	0.047
Otu175_Bacteroidetes	0.66	0.021	Otu896_Lachnospiraceae	0.54	0.044
Otu783_Prevotella	0.67	0.017	Otu316_Lachnospiraceae	0.56	0.039
Otu332_Coriobacteriaceae	0.78	0.002	Otu580_Bacteria	0.59	0.028
			Otu529_Prevotella	0.59	0.027
			Otu737_Saccharofermentans	0.60	0.025
			Otu613_Lachnospiraceae	0.60	0.023
			Otu782_Bacteroidetes	0.60	0.023
			Otu859_Bacteria	0.61	0.020
			Otu913_Treponema	0.62	0.018
			Otu135_Coriobacteriaceae	0.62	0.018
			Otu476_Firmicutes	0.63	0.017
			Otu273_Firmicutes	0.71	0.005
			Otu692_Bacteria	0.73	0.003
			Otu210_Bacteria	0.81	0.001
			Otu534_Prevotellaceae	0.84	0.001

On d51, negative correlations were observed in microbiota including unclassified *Clostridiales* (Otu555, Otu780), *Prevotella* (Otu475, Otu970, Otu505), and unclassified *Coriobacteriaceae* (Otu551). However, unclassified *Coriobacteriaceae* (Otu332), unclassified *Bacteroidales* (Otu438, Otu175), and *Prevotella* (Otu783) were positively correlated with NEFA.

On d115, the relative abundances of unclassified *Clostridiales* (Otu479), unclassified *Prevotellaceae* (Otu238), and unclassified *Bacteroidetes* (Otu599) decreased when serum NEFA increased. Rumen microbiota including unclassified *Lachnospiraceae* (Otu896, Otu316, Otu613), unclassified *Coriobacteriaceae* (Otu135), unclassified *Prevotellaceae* (Otu529, Otu534, Otu957), *Clostridiales* (Otu555), *Saccharofermentans* (Otu737), *Bacteroidetes* (Otu782), and *Treponema* (Otu913) linearly increased with increasing NEFA.

## Discussion

This study was conducted to investigate changes within the rumen microbiota in gestating and lactating ewes that grazed tall fescue with different levels of endophyte infection. The toxicity (ergovaline) concentration in tall fescue was affected by seasonal effects, which is consistent with a previous study (6), suggesting that grazing at different growing seasons may have varying impacts on animals due to changing ergovaline intake. We observed that the fescue endophyte may negatively influence the health of lactating ewes more so than gestating ewes. Significant changes in serum NEFA and rumen microbial diversity were found at the end of lactation. A consortia of microbiota were linked to levels of endophyte-infection in gestation and lactation periods, and the microbiota responded to the step-wise increase in mycotoxins from the tall fescue. Moreover, the correlation between rumen bacteria and important reproductive parameters (serum PRL and NEFA) was measured that allowed for a better understanding of the host mechanism involving mycotoxin degradation.

Feed with mycotoxins or other potentially harmful substances have been shown to influence the rumen microbiota (16). In the current research, toxins in tall fescue affected alpha-diversity measures such as the number of observed OTUs and Shannon diversity index. Based on these results, there were no differences between d1 and d51, however, HA had greater alpha diversity than MA on d115. Similarly, beta diversity analysis also showed distinct clusters of microbial community structures between HA and MA groups on d115. We speculate that the lack of differences between d1 and51 could be explained by sheep consuming a forage that contained lower levels of toxins, allowing rumen bacteria to detoxify the mycotoxins. However, by d115, toxin concentrations had increased in both treatments resulting in significant impacts on alpha diversities. In a more recent study, Mote et al. (8) found that cattle grazing toxic tall fescue for 28 days (total ergot alkaloid levels 2357.7 ± 19.70 μg/kg) had a tendency to change the overall fecal microbiota community structure. Regarding the concentration of total ergot alkaloid in HA on May 25, 2016 (d114, 3,130 μg/kg), it was perhaps not surprising that significant results on d115 were observed compared with the results on d51.

The random forest algorithm was performed to classify the OTUs differentiating MA and HA treatments between d51 and d115, respectively. In gestation and lactation, only two shared OTUs differentiating high and moderate endophyte infection were observed. One of them, unclassified *Coriobacteriaceae* (Otu269), was enriched in the HA ewes at both time points. Importantly, it has been observed that members of the *Coriobacteriaceae* family can degrade mycotoxins to non-toxic metabolites (29). It was also consistently observed that OTUs associated with *Coriobacteriaceae* were more abundant in HA ewes and therefore could be viewed as an indicator of their contributions to ergovaline detoxification. Likewise, OTUs classified as *Veillonellaceae* and *Lachnospiraceae* were also observed with high abundances in HA ewes, which is in line with a recent study where high abundances of these bacteria were also found in cattle grazing toxic endophyte-infected tall fescue (8). Bacterial community structure has been shown to be affected by other mycotoxins (i.e., deoxynivalenol or fumonisin) in previous studies (30, 31). Therefore, these three rumen bacterial families may be associated with mycotoxin degradation. *Lachnospiraceae* can produce volatile fatty acids (VFA), whose metabolism may be influenced by changes in the bioavailability of the primary fermentation metabolites by mycotoxins (30, 32). Foote et al. (33) confirmed that ergovaline reduced rumen VFA concentrations, which provides further support that intake of highly endophyte-infected tall fescue impacts nutrient bioavailability, and potentially absorption, along with the resident microbiota (34). Moreover, members of the *Prevotella* genus and *Clostridiales* order relatively abundant in MA on d115 are reported as ergovaline degraders (17). Increased relative abundance of *Prevotella* were also observed when animals consumed another metabolic mycotoxin (deoxynivalenol) associated with a *Fusarium* fungi (35). Evidence from an *ex vivo* study demonstrated that *Prevotellaceae* and *Clostridiaceae* strains are not heavily involved in ergovaline degradation (17). We assumed that these two bacterial taxa associated with ergovaline degradation were affected by mycotoxin concentration. A hypothesis is that *Prevotellaceae* and *Clostridiaceae* can degrade ergovaline when its concentration was low, but these bacteria's activity decreased in environments containing high ergovaline concentration. Therefore, the concentration of ergovaline in tall fescue influences community structure of the rumen microbiota, with bacteria (e.g., *Coriobacteriaceae, Veillonellaceae*, and *Lachnospiraceae*) in high abundance in HA potentially playing roles in endophyte degradation.

Prolactin is known to play an important role for both lactation and mammogenesis (36). Prolactin homeostatic levels are sensitive to tall fescue toxins and are a measure of the degree of tall fescue mycotoxicosis (37). Since ergovaline has a ring structure similar to the major prolactin inhibitor (dopamine), this may significantly affect serum prolactin concentration (16). In the current study, serum prolactin levels were not statistically different between treatments; however, numerically lower values were observed in MA treatment on d115. Duckett et al. (38) found that serum PRL levels did not differ between ewes fed endophyte-infected tall fescue seed (0.8 μg of ergovaline/g diet DM) and free-infected seed on d 30 of gestation. Yet, significantly lower PRL levels occurred in ewes exposed to ergot alkaloids on d50 and d130 of gestation compared to an endophyte-free group (38). Additionally, another study did not find serum prolactin differences in goats grazing endophyte-infected tall fescue (87.5–95.8% infection) (39). It was speculated that the change of serum PRL was dependent on the concentrations of total ergot alkaloid in forage and the intake of tall fescue, as well as the ability of the rumen microbiota to degrade ergovaline. Consequently, we compared the correlation of PRL concentrations with the microbiota exposed under different levels of mycotoxins, which, in turn, could reflect the ability of rumen microbiota to degrade ergovaline. *Lachnospiraceae* exhibited a negative relation with PRL concentration in both gestation and lactation periods. Additionally, we observed that *Clostridiales* and *Coriobacteriaceae* were negatively correlated to serum prolactin concentration. Moreover, we found OTUs related to *Prevotella* that occurred in high abundance in MA were negatively correlated with PRL. *Prevotella* species were shown to induce a ruminal environment favorable to *Prevotella* and other hyper-ammonia-producing bacteria, potentially beneficial by reducing the prevalence of fescue toxicosis (16). Therefore, the intake of toxin could likely influence the rumen microbiota and ultimately impact serum prolactin.

There were several limitations of this study. First, while the baseline can be used as a control for endophyte infection, there was no control group that was exposed to non-infected tall fescue. However, endophyte-free tall fescue is not persistent in this area. Also, pastures seeded to newer non-toxic, novel-endophyte infected tall fescue are not located on the USDA station. Use of another forage as a control would confound the information by having different forages and different levels of toxicity. Second, this study did not measure the rumen alkaloid concentrations. Some measures of intake (based on weight gain), rumen volume and rate of passage would help estimate the extent of alkaloid degradation. Greater numbers of samples would be needed to measure the alkaloid concentration, but would have resulted in excessive stress on animals that are already stressed to a greater extent because of the toxins. Inserting a stomach tube causes considerable stress on the animal and the sampling days were hot, which would have caused excessive stress. Measuring rumen volume and rate of passage would require the use of pulse-dosed markers such as Yb then repeated and frequent sampling to develop the fecal excretion curve of the inserted marker. This again would have required a greater number of times the animals were handled and would not be allowed by the animal welfare group that oversees the animals at the USDA station.

To date, this is the first study to characterize the rumen microbiota and its relationship with serum prolactin and NEFA in gestating and lactating ewes grazing tall fescue with high or moderate levels of toxic endophyte infection. Since the concentration of total ergot alkaloids increased throughout the study, accurate levels of ergovaline in tall fescue that are shown to influence the rumen microbiota composition and ewes' phenotype cannot be confirmed. However, this study did identify rumen microbial changes affected by moderate and high levels of toxins in endophyte-infected tall fescue. Characteristics of the rumen microbiota associated with serum prolactin and NEFA were additionally identified. High endophyte infection exhibited increased abundances of *Coriobacteriaceae, Lachnospiraceae*, and *Prevotellaceae* families, which could be related with the endocrinology and metabolism of fatty acids. Future studies are needed to accurately identify the concentration of ergovaline significantly altering the rumen microbiota, and to further delineate the association between the rumen microbiota and host physiology.

## Data Availability Statement

The datasets presented in this study can be found in online repositories. The names of the repository/repositories and accession number(s) can be found in the article/Supplementary Material.

## Ethics Statement

The animal study was reviewed and approved by University of Arkansas Institutional Animal Care and Use Committee.

## Author Contributions

KC, JB, and JZ conceived and designed the experiments. SA and JB performed the experiment and collected samples. JC, SP, and JZ performed next-generation sequencing. JC, SA, KC, JB, and JZ analyzed and interpreted the data. JC, SA, KC, JB, KF, SR, JE, and JZ drafted and revised the manuscript. All authors contributed to the article and approved the submitted version.

## Conflict of Interest

The authors declare that the research was conducted in the absence of any commercial or financial relationships that could be construed as a potential conflict of interest.
