# Supplementary material for: Endophyte-Infected Tall Fescue Affects Rumen Microbiota in Grazing Ewes at Gestation and Lactation
Source: Front Vet Sci. 2020 Oct 14;7:544707. doi: 10.3389/fvets.2020.544707 (PMC7591458; doi:10.3389/fvets.2020.544707)
Supplement: Supplementary file 1 [file Data_Sheet_1.docx]

**Endophyte-infected tall fescue affects rumen microbiota in grazing ewes at gestation and lactation**

**Jianmin Chai^1#^, Saleh Alrashedi^1#^, Ken Coffey^1^, Joan M. Burke^2^, Kristina Feye^3^, Steven C. Ricke^3^, Si Hong Park^4^,** **J Lannett Edwards^5^, Jiangchao Zhao^1*^**

^1^Department of Animal Science, Division of Agriculture, University of Arkansas, Fayetteville, AR, USA;

^2^United States Department of Agriculture, Agricultural Research Service, Booneville, AR, USA

^3^Department of Food Science and Center for Food Safety, University of Arkansas, Fayetteville, AR, USA;

^4^Department of Food Science and Technology, Oregon State University, Corvallis, OR, USA;

^5^Department of Animal Science, University of Tennessee, Knoxville, TN, USA;

# these authors contributed equally to this work.

***Correspondence**:

Dr. Jiangchao Zhao

[jzhao77@uark.edu](mailto:jzhao77@uark.edu)

**Supplementary Information**

**Table S1.** Ewe lambing supplement and guaranteed analysis of the mineral supplement offered free-choice to all ewes grazing tall fescue pastures with different levels of infection with *Neotyphodium coenophialum.*

**Table S2.** Monthly temperatures and precipitation for the growing season (February through May) of 2016.

**Table S3.** Serum prolactin (PRL) and non-esterified fatty acid (NEFA) from ewes grazing tall fescue pastures with high (HA) and moderate (MA) *Neotyphodium coenophialum* infection from February through May. Lambing began on d 51 and d115 corresponded to the end of the study when lambs averaged 60 days of age.

**Figure S1.** AUC curve for AUCRF differentiating rumen bacteria from ewes consuming tall fescue infected with moderate (MA) and high (HA) levels of *Neotyphodium coenophialum* on days 1 (d1), 51 (d51), and 115 (d115).

**Figure S2.** Bacteria OTUs determined using random forest differentiating rumen microbiota from ewes consuming tall fescue infected with moderate (MA) and high (HA) levels of *Neotyphodium coenophialum* on days 51.

**Figure S3.** Bacteria OTUs determined using random forest differentiating rumen microbiota from ewes consuming tall fescue infected with moderate (MA) and high (HA) levels of *Neotyphodium coenophialum* on days 115.

**Table S1.** Ewe lambing supplement and guaranteed analysis of the mineral supplement offered free-choice to all ewes grazing tall fescue pastures with different levels of infection with *Neotyphodium coenophialum.*

Ewe lambing supplement Mineral supplement

Component % as fed Component Guaranteed analysis

Barley 37.92% Calcium (max) 18.0%

SBM 10.15% Calcium (min) 15.0%

Cracked corn 41.91% Phosphorus 8.0%

Molasses 7.10% Salt (max) 22.2%

Salt (min) 18.5%

CP 13.8%* Potassium 1.5%

TDN 82.2%* Magnesium 5.0%

Copper (max) 375 ppm

Copper (min) 275 ppm

Iodine 320 ppm

Manganese 2,000 ppm

Zinc 3,500 ppm

Vitamin A 63,500 IU/kg

Vitamin D_3_ 15,875 IU/kg

Vitamin E 340 IU/kg

*Calculated from (NRC, 2007)

ppm = parts per million or mg/kg

IU/kg = international unit per kg.

**Table S2.** Monthly temperatures and precipitation for the growing season (February through May) of 2016

| Items | | February | March | April | May |
| --- | --- | --- | --- | --- | --- |
| Temperature/ °C | Low | 0.0 | 4.7 | 9.7 | 13.4 |
|  | High | 15.0 | 19.5 | 23.2 | 24.9 |
| Total Precipitation/mm | | 46.7 | 164.4 | 138.5 | 79.7 |

**Table S3.** Serum prolactin (PRL) and non-esterified fatty acid (NEFA) from ewes grazing tall fescue pastures with high (HA) and moderate (MA) *Neotyphodium coenophialum* infection from February through May. Lambing began on d 51 and d115 corresponded to the end of the study when lambs averaged 60 days of age.

|  | HA | MA | SEM^a^ | *P* values | | |
| --- | --- | --- | --- | --- | --- | --- |
|  |  |  |  | Treatment | Sampling time | Treatment×Sampling time |
| Serum PRL | | |  |  |  |  |
| d1 | 33.5 | 26.2 | 2.69 | 0.998 | 0.008 | 0.350 |
| d51 | 39.1 | 41.8 | 3.11 |  |  |  |
| d115 | 42.7 | 47.4 | 3.8 |  |  |  |
| Serum NEFA | | |  |  |  |  |
| d1 | 410.4^A^ | 535.4^A^ | 39.3 | 0.838 | <0.001 | 0.038 |
| d51 | 853.5^A^ | 914.3^A^ | 41.3 |  |  |  |
| d115 | 464.5^A^ | 315.0^B^ | 32.00 |  |  |  |

^a^SEM = Pooled standard error of the means.

For serum NEFA, different letters between entries in rows are significantly different between treatments at specific sampling time (*P* < 0.05).

Figure S1


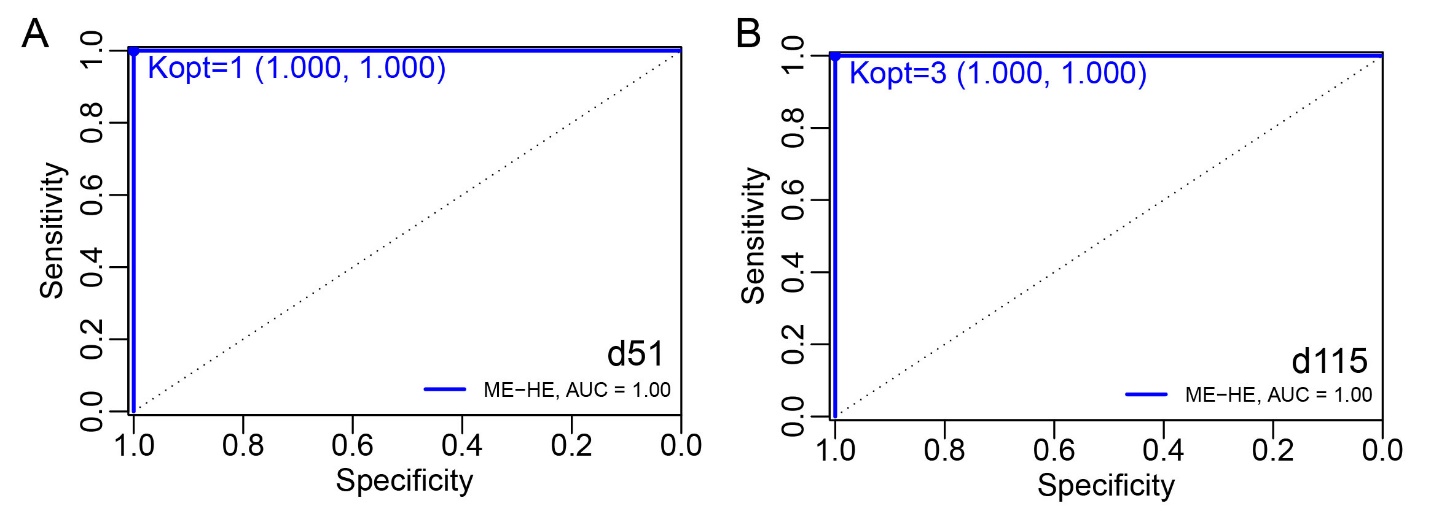


**Figure S1**. AUC curve for AUCRF differentiating rumen bacteria from ewes consuming tall fescue infected with moderate (MA) and high (HA) levels of *Neotyphodium coenophialum* on days 1 (d1), 51 (d51), and 115 (d115).

Kopt meant the number of optimal predictors obtained. The content within parentheses behind Kopt were (specificity, sensitivity).

Figure S2


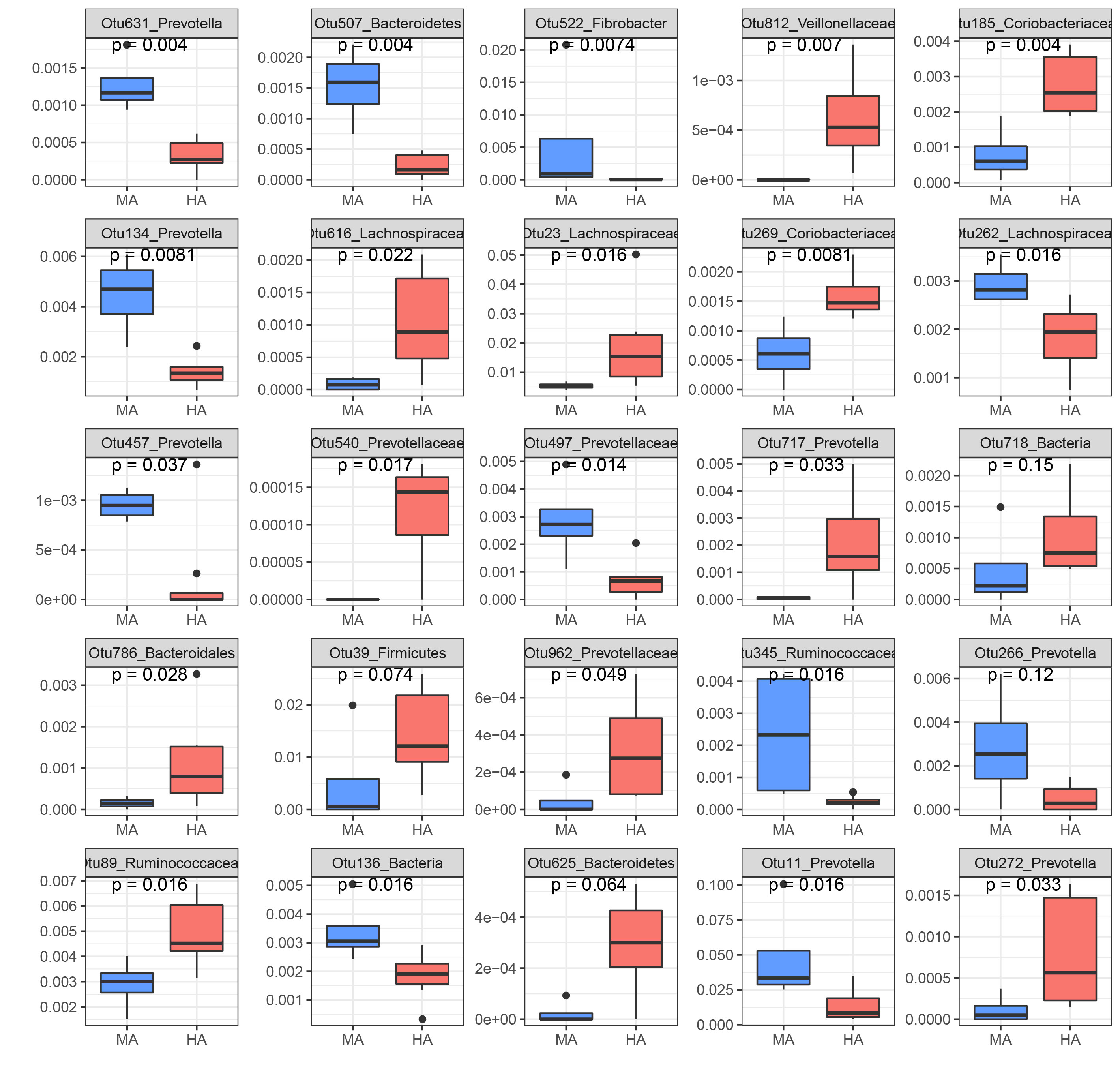


**Figure S2.** Bacteria OTUs determined using random forest differentiating rumen microbiota from ewes consuming tall fescue infected with moderate (MA) and high (HA) levels of *Neotyphodium coenophialum* on days 51.

The rumen microbial data were tested using the Kruskal-Wallis test.

Figure S3


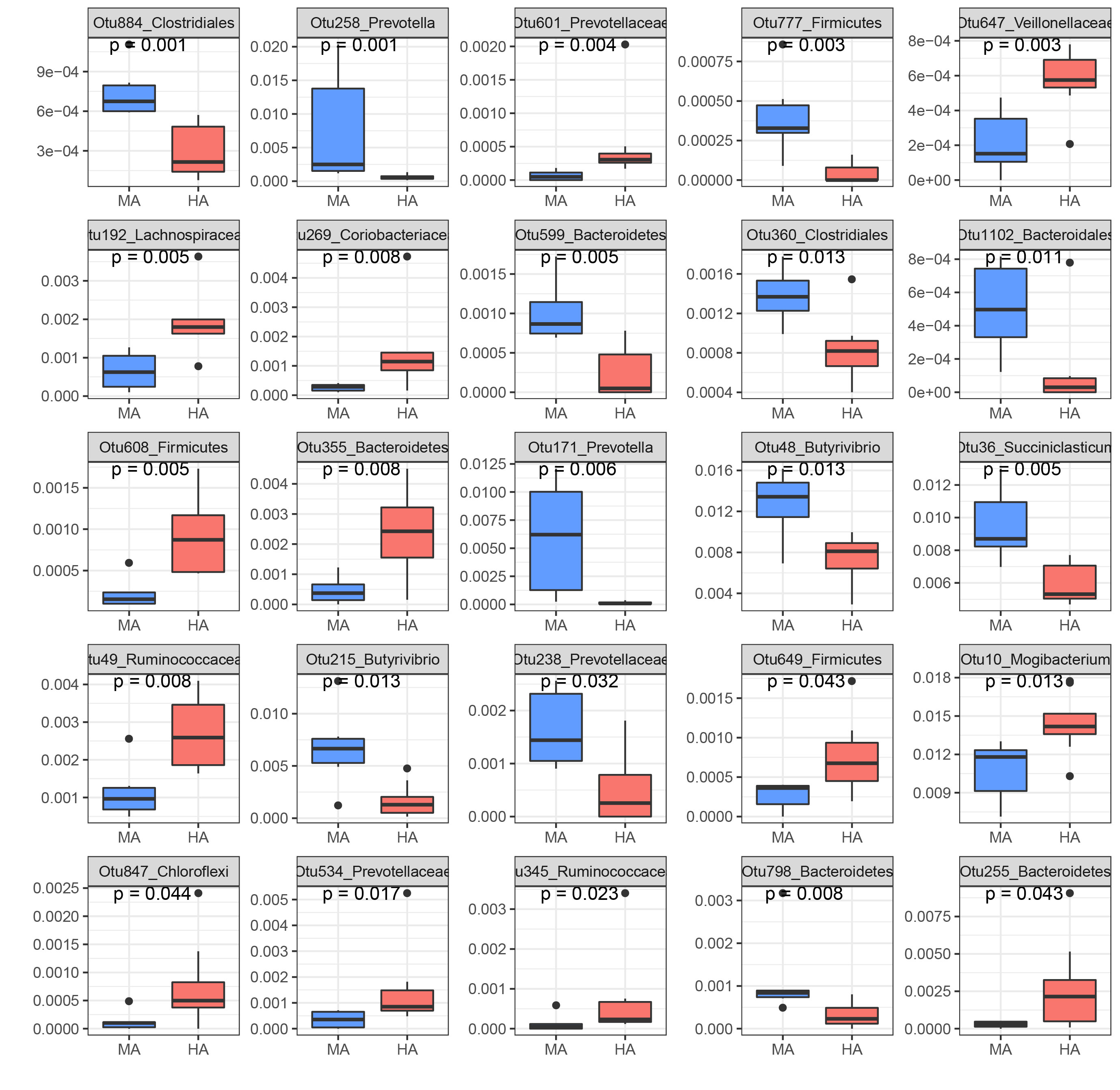


**Figure S3.** Bacteria OTUs determined using random forest differentiating rumen microbiota from ewes consuming tall fescue infected with moderate (MA) and high (HA) levels of *Neotyphodium coenophialum* on days 115.

The rumen microbial data were tested using the Kruskal-Wallis test.
